# *Thermococcus kodakarensis* modulates its polar membrane lipids and elemental composition according to growth stage and phosphate availability

**DOI:** 10.3389/fmicb.2014.00010

**Published:** 2014-01-30

**Authors:** Travis B. Meador, Emma J. Gagen, Michael E. Loscar, Tobias Goldhammer, Marcos Y. Yoshinaga, Jenny Wendt, Michael Thomm, Kai-Uwe Hinrichs

**Affiliations:** ^1^MARUM Center for Marine Environmental Sciences and Department of Geosciences, University of BremenBremen, Germany; ^2^Department of Microbiology and Archaea Center, University of RegensburgRegensburg, Germany

**Keywords:** biomarker, phosphorus limitation, cell carbon quota

## Abstract

We observed significant changes in the elemental and intact polar lipid (IPL) composition of the archaeon *Thermococcus kodakarensis* (KOD1) in response to growth stage and phosphorus supply. Reducing the amount of organic supplements and phosphate in growth media resulted in significant decreases in cell size and cellular quotas of carbon (C), nitrogen (N), and phosphorus (P), which coincided with significant increases in cellular IPL quota and IPLs comprising multiple P atoms and hexose moieties. Relatively more cellular P was stored as IPLs in P-limited cells (2–8%) compared to control cells (<0.8%). We also identified a specific IPL biomarker containing a phosphatidyl-N-acetylhexoseamine headgroup that was relatively enriched during rapid cell division. These observations serve as empirical evidence of IPL adaptations in Archaea that will help to interpret the distribution of these biomarkers in natural systems. The reported cell quotas of C, N, and P represent the first such data for a specific archaeon and suggest that thermophiles are C-rich compared to the cell carbon-to-volume relationship reported for planktonic bacteria.

## Introduction

Nucleic acid and lipid biomarkers have been targeted to investigate the vast microbial populations of both Bacteria and Archaea that are found in natural environments, including the ocean (e.g., DeLong, 2005; Ingalls et al., 2006; Hansman et al., 2009), soils (e.g., Rappé and Giovannoni, 2003; Kreuzer-Martin, 2007), and subseafloor sediments (e.g., Biddle et al., 2006; Teske and Sørensen, 2008). Acting as the boundary between living cells and the environment, lipids have enormous potential to serve as proxies of cell metabolism, particularly in regard to environmental stressors that affect cell division or the exchange of solutes across the cell membrane. Over the last decade, advancements in the study of membrane-associated intact polar lipids (IPLs; e.g., phospholipids and glycolipids) have promoted assessments of microbial biomass and activity in subseafloor sediments (Biddle et al., 2006; Lipp et al., 2008; Xie et al., 2013), taxonomic distributions and factors controlling population diversity (Ertefai et al., 2008; Rossel et al., 2008; Schubotz et al., 2009; Popendorf et al., 2011; Rossel et al., 2011; Bale et al., 2013), microbial production rates based on stable isotope label incorporation (Kellermann et al., 2012; Lin et al., 2012), and adaptations of marine plankton to nutrient stress (Van Mooy et al., 2009), among other applications (e.g., Jaeschke et al., 2012). The responses of bacterial membrane lipids to growth and environmental conditions are well reported (e.g., for reviews see Šajbidor, 1997; Zhang and Rock, 2008); however, the parameters that influence archaeal membrane lipid compositions are not well constrained.

To date, controlled investigations of the core lipids that compose archaeal membranes have only been reported for a few archaea, in response to either temperature, growth stage, salinity, or pH (e.g., Kramer and Sauer, 1991; Morii and Koga, 1993; Macalady et al., 2004; Uda et al., 2004; Lai et al., 2008; Matsuno et al., 2009; Boyd et al., 2011); and even fewer studies have investigated associated changes in IPL composition (e.g., Nicolaus et al., 1989; Uda et al., 2001; Shimada et al., 2008). Furthermore, no study has empirically quantified cellular quotas of carbon (C), nitrogen (N), phosphorus (P), or IPLs of a specific archaeon. Our goal was to investigate changes in the elemental and IPL compositions of a model organism, *Thermococcus kodakarensis*, which is a hyperthermophilic, anaerobic, obligately heterotrophic archaeon that can grow respiratively by sulfur reduction or fermentatively on pyruvate or amino acids. Originally isolated from sediment and water from a solfatara (102°C, pH 5.8, Morikawa et al., 1994), *T. kodakarensis* has been widely studied because of its high growth temperature and fast doubling time, and was previously shown to increase its abundance of glycerol-dibiphytanyl-glycerol tetraethers (GDGTs) relative to archaeol (AR) core lipids in the cell membrane in response to increased growth temperature and as populations entered stationary growth phase (Matsuno et al., 2009). We sought to expand on the findings of Matsuno et al. (2009) by further identifying changes in IPL biomarkers associated with population transition to stationary phase, with the expectation that modifications of the archaeal membrane would be more sensitively recorded in the IPL pool compared to core lipids. Preliminary investigations by our group indicated that the *T. kodakarensis* membrane comprised primarily phospholipids; thus, we also sought to monitor phospholipid production by *T. kodakarensis* in response to reduced phosphate concentration in growth media. These responses have implications for the application of IPLs as biomarkers of archaeal activity in the environment and provide a framework to consider the physiological roles of IPL headgroups.

## Materials and methods

### Organism and growth conditions

*T. kodakarensis* JCM 12380 was routinely cultivated in modified JCM medium 280 (http://www.jcm.riken.jp/cgi-bin/jcm/jcm_grmd?GRMD=280&MD_NAME), in which elemental sulfur was replaced with 5 g L^−1^ sodium pyruvate as the energy source, and yeast extract and tryptone were provided at 5 g L^−1^ (Atomi et al., 2004). Wolfe's trace mineral solution was prepared after Wolin et al. (1963), except without chelating agent and including 0.28 g L^−1^ (NH_4_)_2_Ni(SO_4_)_2_.6H_2_O, and 0.01 g L^−1^ of each Na_2_WO_4_.2H_2_O and Na_2_SeO_4_. In order to investigate the effect of altered phosphate concentrations on the growth and lipid content of *T. kodakarensis*, standard medium was first modified to reduce complex organics to an absolute minimum (complete omission of tryptone and only 0.25 g L^−1^ yeast extract; hereafter referred to as reduced-Org) and then further modified by complete omission of added phosphate salts (i.e., reduced-Org&P). The resulting control, reduced-Org, and reduced-Org&P media contained P concentrations of 3.6, 1.7, and 0.04 μmol P L^−1^.

Usually, cultivation of *T. kodakarensis* was at 85°C in a 21-L bioreactor containing 15 L of liquid and a 2-bar nitrogen headspace. Gassing of the bioreactors at ~250 ml min^−1^ was started during exponential phase growth. Samples (1–2 L) of *T. kodakarensis* cells grown on standard medium were collected at exponential and stationary growth phases from each type of growth medium; the data presented represent averages from two or three replicate bioreactors (Table 1). Harvested cells were pelleted by centrifugation (13000 *g*, 30 min, 4°C) and stored at −20°C and lyophilized before lipid extraction.

**Table 1:** **Average ± standard deviation of elemental and IPL compositions of *T. kodakarensis***.

**Growth media**	**Harvest**	***n***	**Growth rate (*d*^−1^)**	**Cell diameter (μm)**	**fg IPL cell^−1^**	**fg C cell^−1^**	**fg N cell^−1^**	**fg P cell^−1^**	**C:N**	**C:P**	**GDGT (%)**
Control	Expo	3	0.8 ± 0.2	nd	0.62 ± 0.11	156 ± 41	42 ± 12	2.9 ± 1.4^*^	4.3 ± 0.1	119 ± 31^*^	5 ± 5
	stat	2	0.0 ± 0.1	1.24 ± 0.02	0.58 ± 0.28	288 ± 150	77 ± 38	7.3 ± 3.6	4.3 ± 0.3	117 ± 32	10 ± 8
Reduced-org	Expo	2	0.6 ± 0.2	nd	0.96 ± 0.10^a^	72 ± 14	16 ± 1.4	nd	5.3 ± 0.5^a^	nd	3 ± 4
	stat	3	0.1 ± 0.1	1.05 ± 0.02^a^	1.18 ± 0.27	65 ± 22^a^	10 ± 8.7^a^	nd	6.8 ± 1.5	nd	5 ± 1
Reduced-Org&P	Expo	3	1.0 ± 0.2	nd	1.37 ± 0.41^a^	87 ± 25	20 ± 6^a^	2.1 ± 1.0	5.1 ± 0.3^a^	113 ± 22	11 ± 1^b^
	stat	3	0.0 ± 0.0	1.08 ± 0.03^a^	0.82 ± 0.31^c^	47 ± 17^a^	8 ± 2^a^^,^^c^	0.8 ± 0.6^a^	6.5 ± 0.8^a^^,^^c^	187 ± 100	14 ± 4^b^

Significant differences are reported for comparisons to corresponding exponential (expo) or stationary (stat) cells cultured in control (a) or reduced-Org media (b), or to exponential cells harvested from reduced-Org&P media (c); nd, no data. The estimates of cell P quota for exponential cells in control media were determined for two replicate samples, as indicated by the asterisk (^*^).

Cells were routinely observed with an Olympus BX 60 phase contrast microscope with an oil immersion objective UPlanFl 100/1.3. Growth was followed by direct cell counting using a Thoma-chamber (depth: 0.02 mm; Marienfeld, Lauda-Koenigshofen, Germany). Phase contrast microscopy was used to determine the diameter of cells of *T. kodakarensis* grown in control, reduced-Org, and reduced-Org&P media (*n* = 32, 29, 46, respectively) using an Olympus CX31 microscope and Olympus image acquisition.

### Carbon and nitrogen analysis

Aliquots of lyophilized cell pellets (0.5–2 mg) were subjected to hydrochloric acid (HCl) vapor overnight to remove inorganic carbon, then mass percentages of C and N were determined after Owens and Rees (1989) using a ThermoFinnigan Flash Elemental Analyzer 2000 (Bremen, Germany). Cellular quotas of C and N were calculated by determining a cells-per-g dry pellet ratio, and were corrected by linear extrapolation of the C and N composition of pellets obtained from 0.5 L of uninoculated, blank media (i.e., <2 mg C L^−1^; 0.6 mg N L^−1^). C:N values are reported as molar ratios.

### Determination of total cellular, inorganic, and organic phosphorus

Freeze-dried cell material was extracted with 1 mol HCl L^−1^ for 16 h on a rotary shaker at room temperature. The inorganic phosphorus concentration in the extract was determined as ortho-phosphate (PO^3−^_4_) by molybdenum blue spectrophotometry (Hach Lange DR5000), using a protocol modified after Hansen and Koroleff (1999).

For total P analysis, freeze-dried cell material was amended with 0.5 mL of 0.1 mol MgSO_4_ L^−1^ and combusted for 3 h at 550°C (cf. Solórzano and Sharp, 1980). After cooling, the ash was extracted with 1 mol HCl L^−1^ for 16 h on a rotary shaker at room temperature. The total phosphorus content of the extract was determined directly by inductively-coupled plasma optical emission spectrometry (ICP-OES, Varian Vista Pro, radial plasma observation).

Cellular organic phosphorus was determined as the difference between total cell phosphorus and inorganic phosphorus; these values were not determined for cells harvested from reduced-Org media because the large amounts of inorganic P that precipitated increased the error of the organic P estimate (see below). C:P values are reported as molar ratios.

### Extraction and analysis of intact polar lipids

IPLs were extracted and quantified according to Sturt et al. (2004) and Lipp et al. (2008) with slight modifications. Briefly, cell pellets were first lyophilized and weighed. Dry cell material (0.02–0.25 g) was combined with pre-combusted sand (2 g) and extracted four times with a methanol/methylene chloride/trichloroacetic acid buffer (2:1:0.8 v/v) using a sonication probe (Bandelin Sonoplus Model HD2200; 5 min, 0.6 s pulses, 200 W). A total lipid extract (TLE) was prepared after phase separation of the extraction buffer. TLEs were dried under a stream of N_2_ gas and resuspended in 1 mL methylene chloride/methanol (5:1). Aliquots of 10 μL were analyzed by tandem high pressure liquid chromatography mass spectrometry using a ThermoFinnigan Surveyor HPLC system coupled to a ThermoFinnigan LCQ Deca XP Plus ion trap mass spectrometer (ion trap-MS) equipped with an electrospray ionization source (San Jose, CA, USA). The most abundant ions identified after the initial scan from 500 to 2000 Da were selected and fragmented up to two times in an ion trap (i.e., MS/MS). The column, solvents, gradients, and conditions were the same as described by Sturt et al. (2004).

IPL ions were identified based on fragmentation patterns as outlined by Yoshinaga et al. (2011) and quantified relative to the response of a phosphatidyl choline diacyl glycerol (PC-DAG) internal standard. Response factors of 2.4 for ARs and 5.9 for GDGTs were applied, based on the averaged relative responses of an AR linked to a phosphatidyl ethanolamine headgroup and a GDGT linked to one glycosidic and one phosphatidyl glycerol headgroup, respectively (0.5–25 ng; *n* = 7; Avanti Polar Lipids, USA). IPLs quantified for each sample were normalized to the number of cells extracted and are presented as cellular quotas (as above). Selected samples were further examined to determine high precision masses of IPLs after injection of the TLE into a Dionex Ultimate 3000RS UHPLC system equipped with an ACE3 C_18_ column (MZ Analysentechnik, Mainz, Germany) and coupled via an electrospray ionization source to a Bruker maXis high resolution quadrupole time-of-flight mass spectrometer (Q-TOF-MS), after Wörmer et al. (2013).

### Statistical analyses

#### Simpson diversity index (D)

The Simpson Diversity Index, typically used to compare species diversity, was applied to IPL data to identify the IPL diversity of each sample, such that:

$$D=1-\sum_{n=1}^{20}{\left(\text{relative abundance}\right)}^{2}$$

The value of the Simpson Diversity Index (*D*) ranges from 0 to 1, with a value approaching 1 representing high IPL diversity and a value of zero representing no diversity.

#### Principal component analysis (PCA)

Changes in the relative abundance of individual IPLs between samples were examined by PCA to further assess IPL variability. This analysis generated principal component (PC) coefficients for each individual IPL and PC scores for each sample, which provided metrics to compare IPL diversity with corresponding data derived for each sample.

#### Significance tests

A homoscedastic student's *t*-test was used to compare data derived from cells grown in different media; a paired student's *t*-test was used to compare data from exponential and stationary phase cells harvested from the same media. Correlations between various parameters were assessed by the two-tailed probability associated with the Pearson correlation coefficient and sample size. Differences were considered significant at *p* < 0.05.

## Results

### Cell growth and harvest

**Figure 1:**
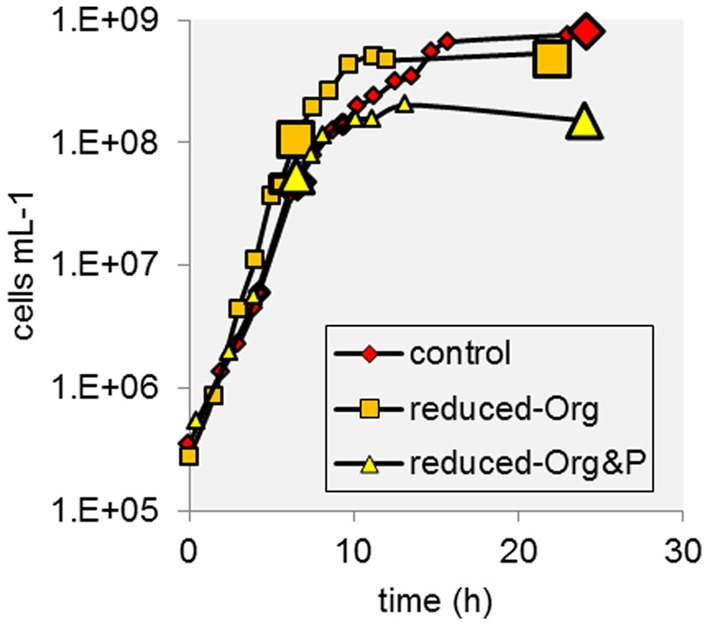
**Example growth curves of *T. kodakarensis* from each treatment**. Sample harvests at exponential and stationary phase are indicated by enlarged data points.

*T. kodakarensis* exhibited typical exponential growth in all media (Figure 1). Cells grown in control media reached maximum densities ranging from 6.5 to 8.1 × 10^8^ cells ml^−1^, whereas this range was typically lower in reduced-Org&P media (0.9 to 1.5 × 10^8^ cells ml^−1^) and also more variable in reduced-Org media (0.3 to 4.8 × 10^8^ cells ml^−1^). The pH was similar for all culture media and exhibited the same trend, decreasing from ~7.5 to ~6.5 during the incubations. The diameter of cells grown in the control media and harvested at stationary phase were on average 1.24 ± 0.02 μm, and significantly larger than those harvested in reduced-Org (1.05 ± 0.03 μm; *p* < 0.001) or reduced Org&P media (1.08 ± 0.02 μm; *p* < 0.001) at stationary phase.

### Cellular elemental composition

Cells cultured in different media contained significantly different elemental composition (Table 1). C, N, and P quotas of stationary phase cells were lower when cultured in reduced-Org or reduced-Org&P media compared to control (*p* < 0.05; Table 1). For exponential phase cells, only the N quota of cells grown in reduced-Org&P media was significantly different from the control (*p* < 0.05). The C:N ratio of exponential phase cells cultured in control media (4.3 ± 0.1) was significantly lower than corresponding cells harvested from reduced-Org (5.3 ± 0.5; *p* < 0.05) or reduced-Org&P media (5.1 ± 0.3; *p* < 0.05). Stationary phase cells harvested from control media also exhibited a significantly lower C:N (4.3 ± 0.3) compared to those of reduced-Org&P media (6.5 ± 0.8; *p* < 0.05). There were no differences in C or N cell quotas observed between reduced-Org and reduced-Org&P cells.

Cellular P quotas varied significantly between replicate harvests and there were no significant differences in C:P (mol:mol) or N:P (mol:mol) observed between growth stage or culture medium, which typically ranged between 90 and 130 or 20 and 30, respectively. The C:P of cells harvested at stationary phase in reduced-Org&P media ranged up to 300 and were always higher than exponential phase cells harvested from the same bioreactor.

The mass percent of organic carbon (%C) of exponential and stationary phase pellets harvested from reduced-Org media (3.1 ± 2.3% and 5.9 ± 6.6%, respectively) were lower than expected for cells (e.g., ca. 50%; Simon and Azam, 1989). The material pelleted from the reduced-Org media was also heavier than expected for the estimated number of cells harvested and contained about an order of magnitude more phosphorus than all other harvests, such that inorganic P accounted for 10% of the dry mass of pellets obtained from reduced-Org media. This resulted in higher error and precluded calculation of cellular P quotas for these cells.

**Figure 2:**
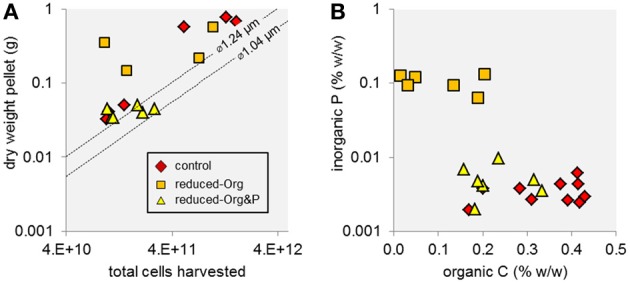
**Mass and elemental composition of *T. kodakarensis* cell pellets harvested from different culture media. (A)** The dry masses of some cell pellets were higher than the projected mass of the total number of harvested cells (dashed lines), which was determined based on measurements of cell diameter (∅ = 1.04 or 1.24 μm) and the cell mass:volume relationship described by Lipp et al. (2008), modified from Simon and Azam (1989), suggesting that non-cellular, insoluble material was harvested in addition to cells. Specifically, harvests of the reduced-Org media and the control media at stationary phase were heavier than the predicted mass of total cells by up to 340 and 550 mg, representing a 22-fold and 5-fold excess of the projected mass of the pellet, respectively. Cells harvested from reduced-Org&P media and from control media at exponential phase were more consistent with predicted mass. **(B)** The reduced-Org harvests contained high amounts of non-cellular material that was rich in inorganic P relative to organic C. For this reason, these samples were excluded in further analysis of cellular P.

Together, the considerably elevated dry mass, low %C, and high inorganic P content of material harvested from the reduced-Org media (Figure 2) suggest that these pellets contained substantial amounts of non-cellular, P-enriched material that precipitated in the absence of soluble organics in the growth media. All media contained iron and zinc (i.e., 1 mg L^−1^ FeSO_4_ · 7H_2_O and 1.8 mg L^−1^ ZnSO_4_ · 7H_2_O) that may have precipitated with phosphate when the media were exposed to an oxygen atmosphere during harvesting. Presumably, decreasing the concentration of complex organics (i.e., yeast extract and tryptone) in the media corresponded to a decrease in organic ligands that kept Fe and Zn in solution; we note that additional chelating agents were removed from the media. This explanation is consistent with (1) the observed mass excesses in material harvested from reduced-Org media and from control media at stationary phase (the control media likely contained more complex organics at exponential phase) and (2) the absence of inorganic precipitates from reduced-Org&P media, with low phosphate amendment.

### IPL identification

At least 20 different IPLs were produced by *T. kodakarensis*, including 9 IPLs that are reported here for the first time (Table 2). All reported IPL ions (M + H and/or M + NH_4_) were detected by ion trap-MS, which was used for quantification and to generate MS/MS fragmentation patterns. IPL identification was based on: **(1)** characteristic mass losses of headgroups during MS/MS fragmentation (cf. Table 2; Yoshinaga et al., 2011), such as phosphatidyl inositol (PI; 242 Da), phosphatidyl ethanolamine (PE; 43 Da), phosphatidyl serine (PS; 87 Da), phosphatidyl glycerol (PG; 74 Da), phosphatidic acid (PA; 80 Da), monoglycosidic (G; 179 Da), and diglycosidic (2G; 342 Da) and/or **2)** elemental formula derived from high precision mass determination after Q-TOF-MS, which were accurate to within 1.8 mDa of the predicted chemical formulae (Table 2). Reference numbers for specific ARs (i.e., IPLs 1–11) and GDGTs (i.e., IPLs 12–20) were generated according to retention time (cf. Table 2).

**Table 2:** **IPLs of *T. kodakarensis*. Novel IPLs are indicated in bold**.

**IPL**	**Core**	**Headgroup**	**Acronym**	Chemical formula^***^	**RT (min)**	**M + H (Da)**	**M + NH_4_ (Da)**	**Reference and/or figure**
1	AR	Phosphatidic acid	PA-AR	^****^	11.9	733.1	*Nd*	Yoshinaga et al., 2011
2		Phosphatidyl glycerol	PG-AR	C_46_H_96_O_8_P	11.8	807.6847	824.7098	Yoshinaga et al., 2011
3		Diglycosidic	2G-AR	C_55_H_112_NO_13_	13.7	nd	994.8122	Yoshinaga et al., 2011
4		Phosphatidyl ethanolamine	PE-AR	C_45_H_9__5_NO_6_P	13.9	776.6894	nd	Yoshinaga et al., 2011
5		Phosphatidyl N-acetylhexoseamine	P-HexNAc-AR	C_51_H_103_NO_11_P	17.3	936.7276	953.8	Ferrante et al., 1986, Figure 4
**6**		68 (Da) + hexoseamine + phosphatidyl inositol	**68 + Hex(NH_2_)-PI-AR**	C_60_H_119_NO_15_P	20.5	1124.8316	nd	Figure 4
7		Phosphatidyl serine	PS-AR	^****^	21.3	820.6	nd	Yoshinaga et al., 2011
8		Phosphatidyl inositol	PI-AR	C_49_H_100_O_11_P	22.3	895.7008	912.7270	Yoshinaga et al., 2011
9		Hexoseamine + phosphatidyl inositol	Hex (NH_2_)-PI-AR	C_55_H_111_NO_15_P	25.4	1056.7698	1073.8	Nishihara et al., 1992
**10**		Hexose + phosphatidyl inositol	**Hex-PI-AR**	C_55_H_110_O_16_P	27.2	1057.7526	1074.8	Figure 4
**11**		Hexoseamine + diphosphatidyl inositol	**PI-Hex(NH_2_)-PI-AR**	C_61_H_1__22_NO_2__3_P_2_	42.9	1298.788	nd	Figure 4
12	GDGT	Phosphatidyl glycerol	PG-GDGT	^****^	13.1	1455.97	1472.03	Yoshinaga et al., 2011
13		Diglycosidic	2G-GDGT	C_98_H_196_NO_16_	14.5	nd	1643.4541	Sturt et al., 2004
**14**	Me-GDGT^*^	Diglycosidic	**2G-Me-GDGT**	C_99_H_198_NO_16_	14.7	nd	1657.477	Figure 3
**15**	diMe-GDGT^**^	Diglycosidic	**2G-2Me-GDGT**	C_100_H_200_NO_16_	14.5	nd	1671.4862	Figure 3
16		Phosphatidyl inositol	PI-GDGT	C_92_H_184_O_14_P	22.4	1544.3443	1561.3706	Jahn et al., 2004
**17**		Hexoseamine + phosphatidyl inositol	**Hex(NH_2_)-PI-GDGT**	C_98_H_195_NO_18_P	26.2	1705.4121	*nd*	Figure 5
**18**		Hexose + diphosphatidyl inositol	**Hex-PI-GDGT-PI**	^****^	33.3	1948.80	*nd*	Figure 5
**19**		Hexoseamine + diphosphatidyl inositol	**Hex(NH2)-PI-GDGT-PI**	^****^	37.9	1947.10	*nd*	Figure 5
**20**		Diphosphatidyl inositol	**PI-GDGT-PI**	^****^	38.9	1785.90	1801.7	Figure 5

* *Methylated GDGT*.

** *Dimethylated GDGT*.

*** Compounds identified based on exact mass and fragmentation patterns.

**** *Identification based on fragmentation patterns only*.

*nd, no data*.

**Figure 3:**
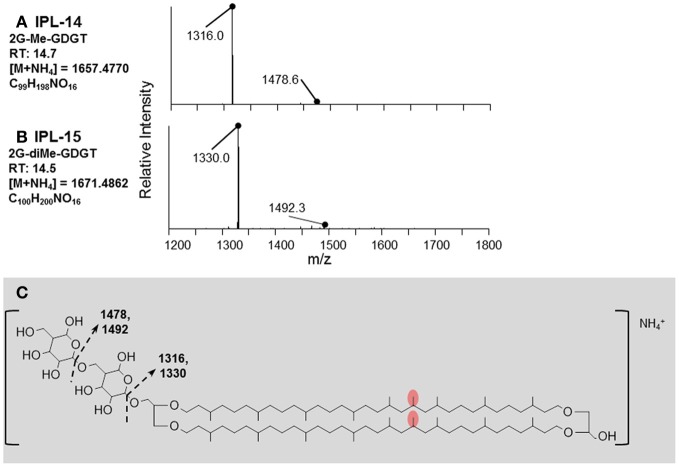
**Identification of diglycosidic IPLs containing methylated and dimethylated GDGT core lipids produced by *T. kodakarensis*. (A)** The retention time, exact mass, elemental formula, and MS/MS fragmentation pattern of 2G-Me-GDGT. **(B)** The retention time, exact mass, elemental formula, and MS/MS fragmentation pattern of 2G-diMe-GDGT. **(C)** The fragments identified in **(A,B)** are explained by the losses of glycosidic headgroups from the core lipid, which has an mass equal to an addition of one or two methyl groups (red ovals; after Knappy, 2010) to the GDGT core lipid (m/z = 1302.3227).

**Figure 4:**
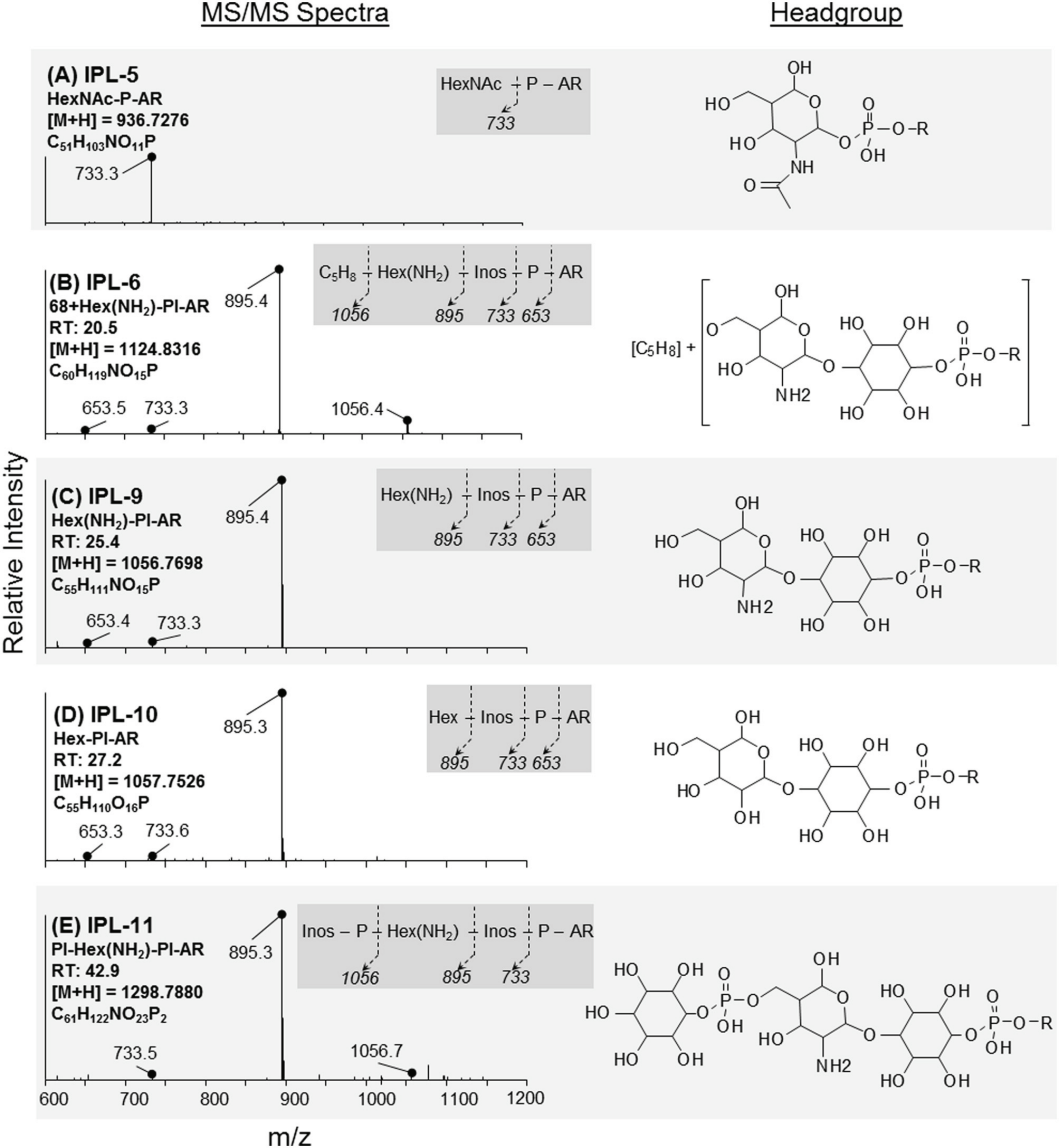
**Identification and tentative structural assignment of novel AR IPLs produced by *T. kodakarensis***. Fragmentations, retention times, and parent ion masses of AR IPLs were identified after ion trap-MS and Q-TOF-MS. MS/MS spectra (left column) revealed major ions that corresponded to fragmentation of headgroup moieties as denoted in the dark grey box; Inos = inositol. Tentative molecular structures are provided in the right column, where *R* = archaeol. **(A)** IPL-5; the MS/MS spectrum is consistent with the P-HexNAc headgroup reported by Ferrante et al. (1986). **(B)** IPL-6; mass fragments of 895 and 1056 Da are consistent with PI-AR and Hex(NH_2_)-PI-AR (see below); the remaining 68 Da mass loss was attributed to a C_5_H_8_ group, which was supported by exact mass determination (cf. Table 2). **(C,D)** IPL-9 and IPL-10; the predicted elemental formula indicated that the 161 Da loss of IPL-9 was likely a hexose substituted with an amino group [i.e., hexoseamine; Hex(NH_2_)], whereas IPL-10 contained a hydroxylated hexose moiety (Hex). **(E)** IPL-11; the 1056 Da daughter fragment is consistent with that of IPL-9 and the loss of a PI moiety. The elemental formula determined after exact mass determination the presence of 1 N atom and 2 P atoms in this IPL.

**Figure 5:**
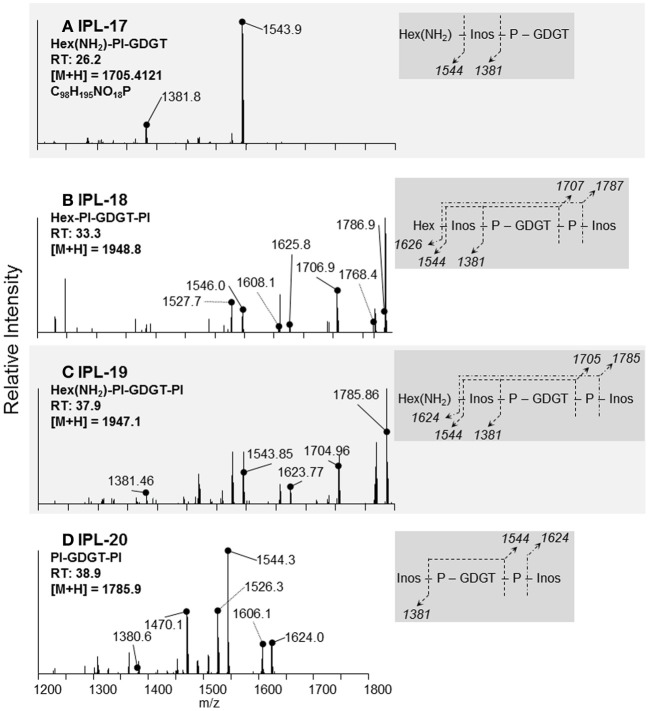
**Identification and tentative structural assignment of novel GDGT IPLs produced by *T. kodakarensis***. IPLs containing a GDGT core lipid were deduced via fragmentation patterns, retention times, and parent ion masses identified after ion trap-MS and Q-TOF-MS. Expected mass losses of known headgroup moieties are illustrated in the dark gray box and correspond to daughter ions identified in the MS/MS spectra. The daughter ions labeled with dotted lines in the MS/MS spectra refer to a loss of water (18 Da) from fragments identified in the dark gray box. **(A)** IPL-17; the 161 Da loss and elemental formula are consistent with the loss of a Hex(NH_2_) or Hex, as in Figure 4C, yielding the PI-GDGT daughter ion (m/z = 1544 Da). **(B,C)** IPL-18 and IPL-19; the similar fragmentation patterns and mass difference of parent ions of these IPLs are parallel to the headgroups identified in IPLs-9 and 10 (Figures 4C,D). In the case of IPL-19, the 1705 Da daughter ion is consistent with IPL-17 after the loss of a PI moiety. **(D)** IPL-20; the 1544 Da fragment identifies PI-GDGT after the loss of one PI moiety.

IPLs exhibiting parent or daughter ions of m/z 895, with MS/MS fragment ions of m/z 733 and 653, were interpreted as PI, which is consistent with that commonly observed in thermophilic Archaea (e.g., Daiyasu et al., 2005; Koga and Nakano, 2008; Oger and Cario, 2013), although the specific stereochemistry of the hexose moiety was not confirmed in the current study. The novel 2G-methylated GDGT (2G-Me-GDGT; IPL-14) and 2G-dimethylated GDGT (2G-diMe-GDGT; IPL-15) were identified by exact masses after Q-TOF-MS and MS/MS fragmentation patterns (Table 2; Figure 3), which were consistent with the Me-GDGT core lipids reported by Knappy et al. (2009) and Knappy (2010). These methylated core lipids (i.e., 1316.3Da and 1330.4 Da, respectively) were generated via removal of the 2G headgroups after mild acid hydrolysis (Meador et al., unpublished data) and co-eluted with GDGT (1302.2 Da) during normal phase chromatography, further confirming that the additional methyl group was located in the biphytanyl chain and not in the glycerol moiety (Zhu et al., 2014). The arrangements of headgroup moieties of other IPLs were tentatively deduced from fragments obtained by MS/MS spectra (Figures 4, 5). The predicted elemental formulae based on exact mass information were consistent with the headgroups deduced from MS/MS spectra; however, we did not apply additional structural elucidation methods (e.g., NMR) and cannot confirm the exact structures of these IPLs. For one compound [i.e., 68+Hex(NH_2_)-PI-AR], the PI-AR daughter ion was identifiable after fragmentation but the associated mass loss could not be attributed to an acknowledged headgroup moiety; the mass of the loss (Da) is thus reported together with the daughter fragment (Table 2; Figure 3B). Of the 9 novel IPLs produced by *T. kodakarensis*, 7 contained a PI headgroup linked to additional hexose (Hex), hexoseamine [Hex(NH_2_)], and/or PI moieties, which were adjoined to either AR or GDGT core lipids (Table 2; Figures 4, 5).

Molecular ions of core lipids were also observed after Q-TOF-MS (not shown). The relative abundance of AR (93 ± 3%; m/z 653.6806) was much greater than GDGT (6 ± 3%; m/z 1302.3227), similarly to that observed for IPLs. Minor core lipids were also detected, including a GDGT with one pentacyclic ring (m/z 1300.3070; <1%), and glycerol dibiphytanol diether with no rings (2 ± 1%; m/z 1246.2965; cf. Liu et al., 2012). Collectively, integrated ion chromatograms of core lipids always represented <10% of the peak area of that of IPLs (data not shown). No IPLs were detected in the TLE extracts of pellets from blank media.

### Cellular IPL inventory

**Figure 6:**
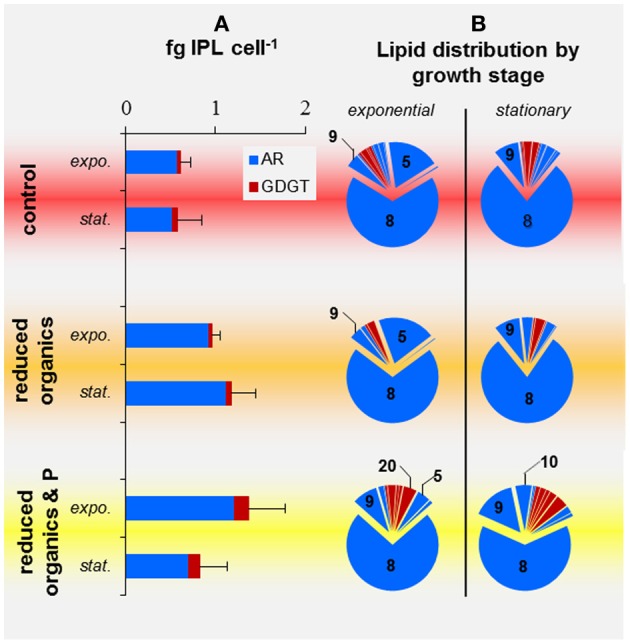
**IPL profiles of *T. kodakarensis* harvested at exponential or stationary phases from various media**. **(A)** Cell quotas of AR (blue) and GDGT (red) IPLs; error bars represent standard deviation. **(B)** Relative abundance of IPLS; those that composed >5% are noted by the respective IPL number provided in Table 2.

Replicate harvests were highly variable in cellular IPL quota, which ranged from 0.38 to 0.74, 0.70 to 1.42, and 0.50 to 1.61 fg IPL cell^−1^ for cells harvested from control, reduced-Org and reduced-Org&P media, respectively (Figure 6A). Cellular IPL quotas of *T. kodakarensis* grown in reduced-Org (0.79 ± 0.10 fg IPL cell^−1^) and reduced-Org&P media (1.34 ± 0.41 fg IPL cell^−1^) were significantly higher than control cells at exponential phase (0.59 ± 0.08 fg IPL cell^−1^; *p* < 0.05; Figure 6A). Stationary phase cells cultured in reduced-Org&P media were consistently depleted in IPLs by 30–50% compared to exponential phase cells cultured in the same bioreactor (*p* = 0.05), while stationary phase cells in other treatments were unaffected.

### IPL composition and diversity

Phospholipids consistently dominated over glycolipids in all cells investigated in the current study, and phosphatidyl inositol archaeol (PI-AR; IPL-8) was the most abundant IPL in all harvests (55–88% of IPLs, Figure 6B). Glycolipids composed only a minor fraction of membrane lipids in *T. kodakarensis* and were primarily observed in the exponential growth phase of cells cultured in the control media, where they accounted for ≤14% of IPLs. No glycolipids were detected in cells grown in reduced-Org or reduced-Org&P media.

**Figure 7:**
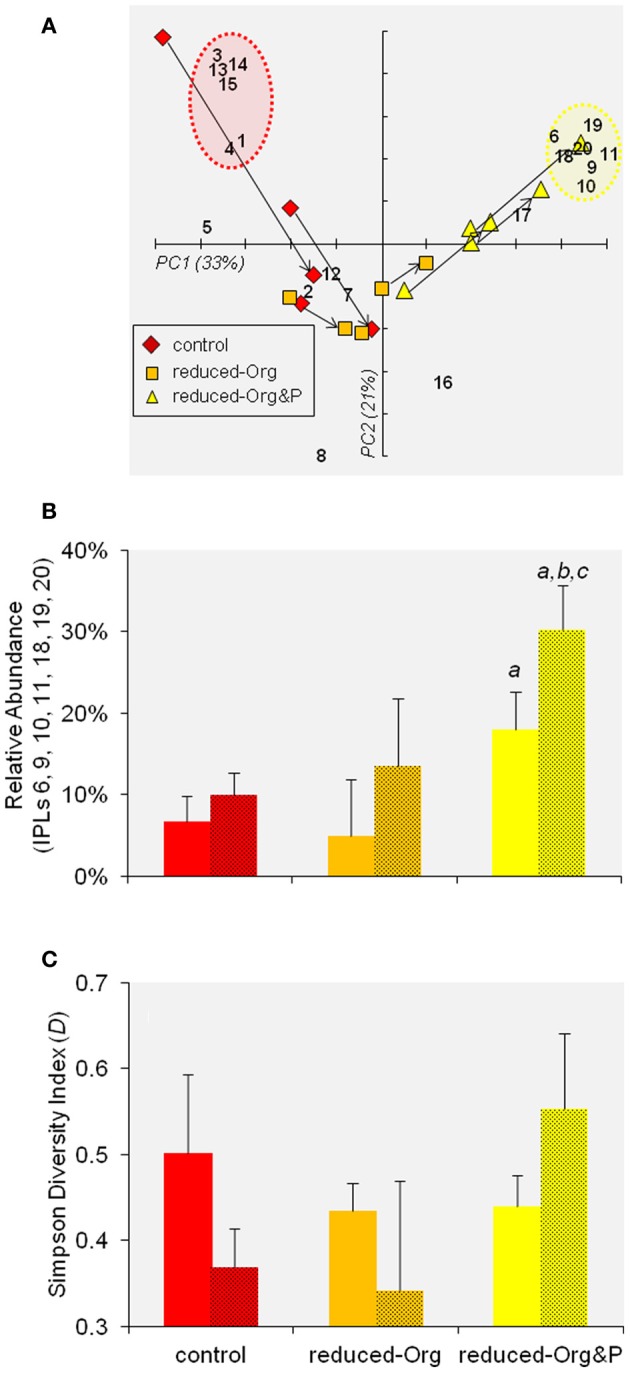
**PCA of IPL distributions**. **(A)** Biplot of PC1 and PC2 of IPL distributions in *T. kodakarensis*. The numbers refer to individual IPLs as described in Table 2. The arrows indicate the progression of cells from exponential to stationary phase within individual bioreactors. The red and yellow circles identify the clusters of IPLs that were relatively enriched in control or reduced-Org&P cells, respectively (refer to Section IPL Composition and Diversity). **(B)** The sum of the relative abundance of IPLs identified by the yellow circle in **(A)** are compared for cells harvested from various culture media at exponential (not shaded) and stationary (shaded) phases. The error bars represent standard deviation and the *a, b*, and *c* symbols represent significant differences (*p* < 0.05) relative to corresponding averages for control, reduced-Org media, and exponential harvests, respectively. **(C)** The average Simpson Diversity Index of *T. kodakarensis* IPLs harvested from the various culture media at exponential and stationary phases (as above); error bars represent standard deviation.

PCA provided an overview of IPL variability between samples, in which the PC1 and PC2 axes explained 54% of the variability in IPL composition and identified clusters of cells cultured in control media (i.e., more negative PC1 scores) and those cultured in reduced-Org&P media (i.e., more positive PC1 scores; Figure 7A) when compared to the average composition. Cells cultured in reduced-Org media clustered near the origin, indicating less pronounced variability in their IPL composition.

#### IPLs and culture media

The IPL composition of cells cultured in control and reduced-Org media were more similar than those cultured in reduced-Org&P media, which contained relatively more GDGTs at both exponential and stationary phases (10 ± 2% and 12 ± 5%, respectively) compared to corresponding cells grown in reduced-Org media (2 ± 4% and 4 ± 1%, respectively; *p* < 0.05; Figure 6). Clusters identified by PCA revealed that control cells were associated with IPLs-1, 3, 4, 13, 14, and 15 (Figure 7A, red circle). One of the exponential phase harvests from control medium exhibited the most anomalous IPL composition within the sample set; this sample scored the highest in both PC1 and PC2 due to the relative enrichment of glycosidic IPLs (e.g., IPLs-3, 13, 14, and 15), which accounted for 14% of total IPLs. The diagnostic IPLs that were relatively enriched in reduced-Org&P cells included IPLs-6, 9, 10, 11, 18, 19, and 20 (Figure 7A, yellow circle). The sum of the relative abundances of these IPLs was significantly higher for cells grown in reduced-Org&P media (*p* < 0.05; Figure 7B) and comprised mostly IPL-9 and IPL-20. IPL-11 was a unique AR that accounted for <1% of IPLs and appeared only in cells grown in reduced-Org&P media. IPL-18 was only observed in stationary phase cells cultured in reduced-Org&P media (>1%) and absent in all other harvests, including exponential phase cells cultured in the same media. Additionally, IPL-5 was significantly depleted in exponential phase cells grown in the reduced-Org&P media (5 ± 2%; *p* < 0.001) compared to the control media (>16%); IPL-5 also accounted for a relatively high percentage of exponential phase cells cultured in reduced-Org media (>14%).

#### IPLs and growth stage

The arrows in Figure 7A depict the progression of IPL composition of cells from exponential to stationary growth phases. The trend for cells grown in control media is toward more positive PC1 scores and more negative PC2 scores. The corresponding trend for cells grown in reduced-Org&P media is toward more positive PC1 and PC2 scores. Cells grown in reduced-Org media exhibited less drastic changes in IPL composition with the progression from exponential to stationary phase; the IPL compositions of these cells were more similar to stationary phase harvests from control media and exponential phase harvests from reduced-Org&P media (Figure 7A), all of which exhibited relatively low lipid diversity. In fact, PC2 score was correlated with the Simpson Diversity Index (*D*), such that samples with more negative PC2 scores comprised relatively fewer IPLs and samples with more positive PC2 scores exhibited higher IPL diversity (*p* < 0.001). The arrows in Figure 7A thus illustrate a decrease in IPL diversity as *T. kodakarensis* cultured in control media progressed from exponential to stationary phase (Δ*D* = −0.12 ± 0.13), and conversely, an increase in IPL diversity for cells cultured in reduced-Org&P media (Δ*D* = 0.11 ± 0.06; Figure 7C).

When considering specific IPLs, only IPL-5 varied significantly between exponential and stationary phase cells cultured in control media, decreasing from 18 ± 3% to 3 ± 3% (*p* < 0.02). Cells grown in reduced-Org media also contained significantly higher IPL-5 at exponential phase (21 ± 10%) compared to stationary phase (3 ± 1%; *p* < 0.01). In reduced-Org&P media, the relative percentages of IPL-9 and IPL-10 were significantly higher in stationary phase cells (15 ± 2% and 6 ± 1%) relative to exponential phase cells (9 ± 2% and 2 ± 1%, respectively; *p* < 0.02).

## Discussion

Advancements in the application of archaeal IPLs as biomarkers in natural systems (e.g., Schubotz et al., 2009; Rossel et al., 2011) have highlighted the need to establish links between environmental parameters and IPL distributions. We demonstrate variability in the cellular inventories of 20 different IPLs produced by *T. kodakarensis*, 9 of which are characterized for the first time, in response to changes in both culture growth stage and phosphorus supply. Furthermore, the C, N, and P cell quotas of *T. kodakarensis* were also indicative of nutrient stress and represent, to our knowledge, the first such data for a pure culture archaeon.

### Elemental composition

**Figure 8:**
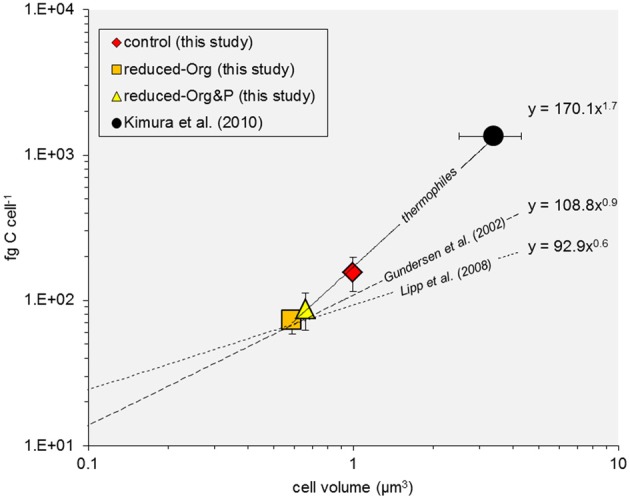
**Cell C:volume power function relationship**. The relationship between measured and modeled values of cell C quota and volume are plotted for thermophiles (solid line), including data derived for *T. kodakarensis* (this study) and a thermophile community (Kimura et al., 2010), and compared to published data for bacteria and archaea (dashed lines; Gundersen et al., 2002; Lipp et al., 2008, after Simon and Azam, 1989).

Ranging up to 220 fg C cell^−l^, the cellular C quotas of *T. kodakarensis* are lower than previous estimates of thermophilic archaea and bacteria (e.g., >1 pg C cell^−1^; Kimura et al., 2010) and approximately an order of magnitude higher than planktonic bacteria (e.g., 10–50 fg C cell^−1^; Lee and Fuhrman, 1987; Fukuda et al., 1998). These differences in cellular C quotas are expected given the relative differences in cell volume [~ 3.4 μm^3^ (Kimura et al., 2010); ~1.0 μm^3^ (this study); 0.03–0.4 μm^3^ (Simon and Azam, 1989)]. However, based on the available C quota data derived from *T. kodakarensis* cultures (this study) and that reported for a natural thermophile community (Kimura et al., 2010), the scaling factor of the C:cell volume power function for thermophiles (i.e., 1.7) appears to be greater than that measured for planktonic bacteria (i.e., <0.9; Gundersen et al., 2002) or modeled for archaea in subseafloor sediments; (Lipp et al., 2008), modified from Simon and Azam (1989); Figure 8. Thermophiles may have thus adapted life strategies for increased relative cellular C content, compared to other populations of bacteria or archaea.

*T. kodakarensis* cultured in reduced-Org or reduced-Org&P media grew at the same rate as when cultured in control media, but cells were ~15% smaller in diameter at stationary phase and contained consistently lower cell quotas of C, N, and P (Table 1). This adaptive strategy increases the surface area to volume ratio, which confers increased nutrient affinity and reduced nutrient demand on smaller cells (e.g., Cotner and Biddanda, 2002). This interpretation is supported by the observed increase in C:N (>5.0) of cells harvested from reduced-Org and reduced-Org&P media compared to control cells (<4.5; *p* < 0.05; Table 1).

### Cellular IPL quotas

*T. kodakarensis* IPL quotas ranged from 0.58 to 1.61 fg IPL cell^−1^ (Figure 6A) and are 80–95% lower than that predicted by the cell membrane:volume relationship described by Lipp et al. (2008), modified from Simon and Azam (1989). A large portion of the cell membrane of *T. kodakarensis* may have been composed of core lipids with no attached headgroup, which would have escaped IPL quantification. However, subsequent analysis of a subset of samples revealed that the *T. kodakarensis* cell membrane comprised only 0.02–0.11 fg core lipid cell^−1^, which represents 9 ± 5% of IPLs (*n* = 19; data not shown) and is insufficient to account for the missing cell membrane mass predicted by the model. Given the lack of available IPL standards, particularly for the newly identified IPLs, we were unable to determine the response factors of individual *T. kodakarensis* IPLs relative to the internal standard; thus, HPLC-MS quantification techniques may have underestimated cellular IPL quotas. It is also possible that the cell membrane of *T. kodakarensis* contains a substantially large portion of S-layer proteins (e.g., Morikawa et al., 1994), which could account for some of the discrepancy between the measured and projected IPL mass. In any case, all samples were subjected to the same quantification biases, thus the significant differences observed between cell IPL quotas and IPL compositions remain valid.

### Factors controlling IPL composition

Phospholipids accounted for the majority of lipids in membranes of *T. kodakarensis* in the current study (Figure 6B). While phosphatidyl inositol (i.e., IPL-8) was the most abundant IPL in all harvests, this IPL exhibited relatively little variability in association with growth stage or P availability. The most diagnostic IPL for *T. kodakarensis* growth stage was an archaeol with a phosphatidyl N-acetyl-hexose headgroup (i.e., IPL-5), which was significantly enriched at exponential phase in cells cultured in control and reduced-Org media (Figure 6B); this IPL may thus serve as a biomarker of rapid cell division in nutrient replete conditions.

**Figure 9:**
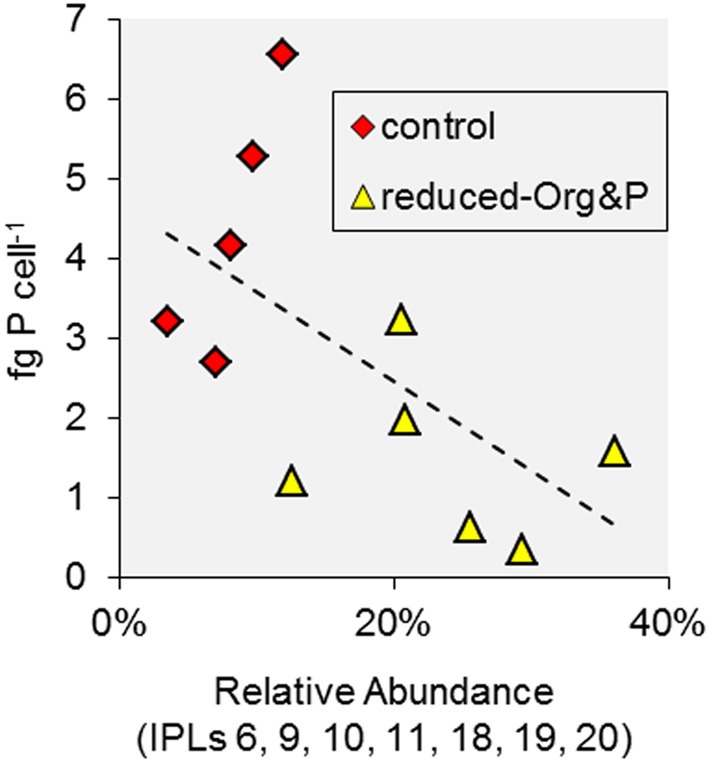
**Factors associated with IPL variability in *T. kodakarensis*.** The relative abundance of IPLs associated with cells cultured in reduced-Org&P media were inversely correlated with cell P quota (*p* = 0.05).

Although *T. kodakarensis* was capable of glycolipid biosynthesis, we observed no glycolipids in cells cultured in reduced-Org&P media, where P amendment was reduced to 1% of the control media. The P concentration of the reduced-Org&P media (i.e., 40 μmol P L^−1^) was similar to that in the hydrothermal sediments, where maximum concentrations are in the range of 5–50 μmol P L^−1^ pore water (Wheat et al., 1996). P concentrations could not be further lowered in the culture media because growth of *T. kodakarensis* required at least 0.25 g yeast extract L^−1^; nevertheless, cells cultured in reduced P conditions exhibited a significantly different lipid composition compared to those cultured in control or reduced-Org media, primarily evidenced by the presence of unique IPLs in these cells (i.e., IPL-6, 9, 10, 11, 18, 19, and 20; Figures 7A,B). These IPLs were inversely correlated with cellular P quota (*p* = 0.05; Figure 9); thus, it appears that similar factors were controlling both cellular P inventory and IPL distribution, such that the unique IPLs containing additional P and/or hexose moieties may serve as biomarkers for P-stress in the environment. Among these, IPL-9 and IPL-20 [i.e., Hex(NH_2_)-PI-AR and PI-GDGT-PI] were the most abundant, accounting for 7–17% and 2–6% of total IPLs in reduced-Org&P cells, respectively, and thus represent the best putative biomarkers for assessing P-limitation.

The Simpson Diversity Index (*D*) of *T. kodakarensis* IPLs exhibited opposite trends with growth stage for cells grown in control or reduced-Org&P media (Figure 7C), which may have been solicited by the exhaustion of alternative growth requirements from the respective media. Given the similarly low IPL diversity exhibited by *T. kodakarensis* in **(1)** reduced-Org media at both exponential and stationary phases, **(2)** control media at stationary phase, and **(3)** reduced-Org&P media at exponential phase (Figure 7C), the variety of IPLs produced by *T. kodakarensis* (i.e., *D*) may have been associated with the exhaustion of organic amendments or the diversity of carbon sources in the growth media. The subsequent increase in IPL diversity of cells in reduced-Org&P media at stationary phase may have then been induced by P-limitation.

### Phosphorus storage in IPLs and geochemical implications

**Figure 10:**
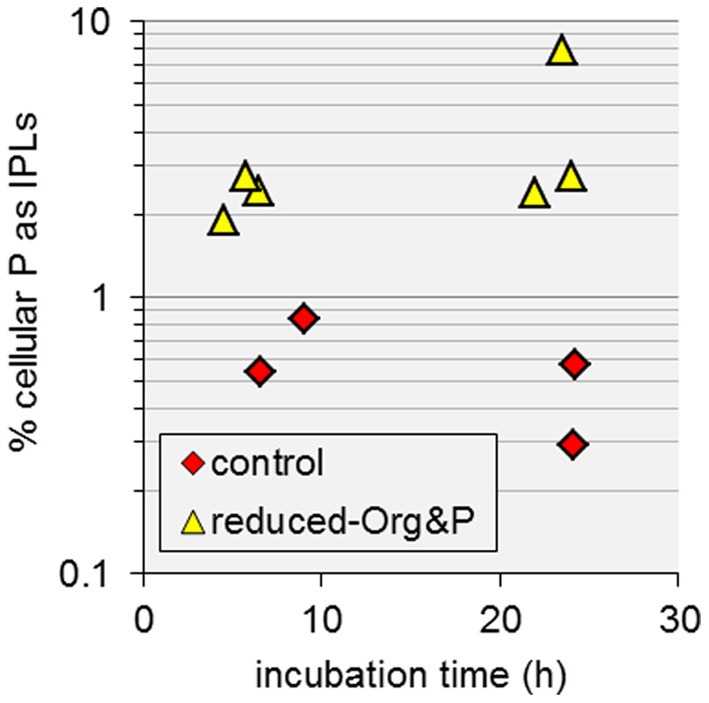
**The amount of cellular P stored as IPLs in *T. kodakarensis***. Cells harvested from reduced-Org&P media (triangles) contained more P as IPLs than control cells (diamonds); however there was large variability in the former, resulting in *p* > 0.05.

*T. kodakarensis* cells grown in reduced-Org&P media exhibited the highest cellular C:P and increased IPL quotas relative to control cells harvested at exponential or stationary phase (Table 1, Figure 6). Additionally, these cells produced unique IPLs containing multiple P atoms (Figure 7B). Consequently, relatively more cellular P was stored as IPLs in cells harvested from reduced-Org&P media (>1.9%) compared to control cells (<0.8%; Figure 10), which is consistent with the reduced nutrient demand for cell machinery (i.e., nucleic acids) expected for smaller cells (see above; Cotner and Biddanda, 2002). The accumulation of P in the cellular IPL pool may thus complement physiological processes that help to sustain nutrient requirements in response to P-limitation in sediment environments. Such a response is opposite to that reported for phosphatidyl IPLs derived from planktonic organisms in the ultraoligotrophic Mediterranean Sea, which decreased in relative abundance with decreasing P concentration in seawater to below 0.1 μmol L^−1^ (Popendorf et al., 2011).

Recently, Marguet et al. (2013) showed that *T. kodakarensis* construct membrane vesicles and nanotubes, which may be used for purposes of cell signaling. In addition, several researchers have also reported heterogeneity in the P composition of minerals in hydrothermal systems (e.g., Fisk et al., 1998), where micro-deposits of P are found adjacent to areas of P-depletion. The observed increases in IPL quotas of cells cultured in reduced-Org and reduced-Org&P media in the current study are in contrast to the observed decreases in cell size, but are consistent with the construction of membrane vesicles and nanotubes by *T. kodakarensis*, which would increase the lipid inventory without producing new cells. The accumulation of P as IPLs (Figure 10) and the production of unique phosphatidyl IPLs with multiple P and hexose-bearing moieties (Figure 7B) may thus be associated with the production of membrane vesicles and nanotubes by *T. kodakarensis* to scavenge P from minerals in hydrothermal environments.

### Conflict of interest statement

The authors declare that the research was conducted in the absence of any commercial or financial relationships that could be construed as a potential conflict of interest.
